# A Topological Multichannel Add-Drop Filter Based on Gyromagnetic Photonic Crystals

**DOI:** 10.3390/nano13111711

**Published:** 2023-05-23

**Authors:** Gangchao Tang, Yuhao Huang, Jianfeng Chen, Zhi-Yuan Li, Wenyao Liang

**Affiliations:** 1 School of Physics and Optoelectronics, South China University of Technology, Guangzhou 510640, China; 2 State Key Laboratory of Luminescent Materials and Devices, South China University of Technology, Guangzhou 510640, China

**Keywords:** gyromagnetic photonic crystals, resonant coupling, add-drop filter, ring resonator

## Abstract

We theoretically proposed a topological multichannel add-drop filter (ADF) and studied its unique transmission properties. The multichannel ADF was composed of two one-way gyromagnetic photonic crystal (GPC) waveguides, a middle ordinary waveguide, and two square resonators sandwiched between them, which can be regarded as two paralleling four-port nonreciprocal filters. The two square resonators were applied with opposite external magnetic fields (EMFs) to support one-way states propagating clockwise and counterclockwise, respectively. On the basis of the fact that the resonant frequencies can be tuned by the EMFs applied to the square resonators, when the intensities of EMFs were the same, the multichannel ADF behaved as a power splitter with a 50/50 division ratio and high transmittance; otherwise, it functioned as a demultiplexer to separate two different frequencies efficiently. Such a multichannel ADF not only possesses excellent filtering performance but also has strong robustness against various defects due to its topological protection property. Moreover, each output port can be switched dynamically, and each transmission channel can operate independently with little crosstalk. Our results have the potential for developing topological photonic devices in wavelength division multiplexing systems.

## 1. Introduction

Topological photonics has attracted extensive attention in recent years due to its unique properties, such as one-way transmission, back-scattering suppression, robust light propagation, and the immunity to various fabrication imperfections [1,2,3,4,5,6,7,8,9]. Photonic Chern topological insulators, as a new state of quantum matter, are known for interesting topological effects in condensed matter physics and potential applications in photonic integrated circuits [10,11,12,13,14,15]. One striking example of topological insulators is one-way topological edge state (TES) at microwave frequencies. By applying an external magnetic field (EMF) to a two-dimensional (2D) gyromagnetic photonic crystal (GPC), TESs are induced as a result of the time-reversal symmetry breaking in analogy with the integer quantum Hall effect [16,17,18,19]. TESs exist at the boundary separating two distinct topological invariants that are characterized by different Chern numbers, which allow photons to propagate in one direction and are highly robust against defects, disorders, and sharp corners. Thus, TESs provide ideal platforms for realizing one-way propagation with a topological protection property, which is a challenge in ordinary waveguides because the backscattering seriously reduces the transmission efficiency and hinders large-scale optical integration.

To date, many fascinating topological phenomena of TESs are explored theoretically and experimentally, such as the cladding-free guiding of topologically protected edge states [20,21,22], the robustness of boundary propagation in amorphous GPC lattices [23,24], and the steering of multiple edge states along domain walls with large Chern numbers [25,26]. Thus, in TESs, a variety of high-performance photonic devices have been constructed on the basis of interesting topological effects and enormous potential in light manipulation, such as topological splitters [27,28], dispersionless slow light states [29,30,31,32], topological circulator [33,34], and topological ADFs [35,36]. Among the aforementioned devices, topological add-drop filters (ADFs) are an important part of the GPC application fields and have triggered considerable attention due to their excellent characteristics, such as low loss, low nonlinear effects, and high transmission coefficients [37,38,39,40,41]. Compared to ADFs constructed by ordinary dielectric PCs, topological ADFs not only have high quality factor and simpler structure without moving specific scatters or introducing heterostructures but they are also robust against fabrication defects [42,43,44]. So far, various types of topological ADFs have been presented and designed, including point-defect microcavities, ring resonators, and multi-cavity-coupled cascade array. Due to its effective frequency selectivity, flexible modulation, and large field enhancement, photonic crystal ring resonator (PCRR) is more popular than other mechanisms. By utilizing the resonant coupling nature between one-way waveguides and the ring resonator, topological PCRR-based ADFs allow optical waves at resonant frequencies in the bus waveguide to transfer to the dropping waveguide via the ring resonator efficiently. With the increasing trend towards wavelength division multiplexing (WDM) systems in the field of large-scale optical integration, topological PCRR-based ADFs have been extensively studied.

Recently, using topological ADFs, a nonreciprocal multichannel power divider for beam splitting or a demultiplexer for frequency separating have been proposed [45,46,47]. Contextually, a natural question appears: Can the circuits and devices based on topological ADFs both realize the function of splitting power flows and separate multiple frequencies? Moreover, can the whole system be flexibly manipulated by adjusting the EMF instead of changing the parameters or dimensions of the structure? These two questions are the main target we need to solve.

In this work, we propose a topological four-port ADF based on the GPC heterostructure, consisting of two one-way waveguides and a square PCRR, and study the influence of the EMF intensity applied to the PCRR on the transmission performance of the ADF. Furthermore, by paralleling two such square PCRRs, we further designed a topological multichannel ADF, which can both realize the functions of power division and frequency separation by adjusting the intensities of EMFs applied to the two PCRRs. Not only can this device possess excellent filtering performance, but also it has good tolerance to various defects, such as metal obstacles and the perturbation of the structural parameters. Our work may provide a deeper understand of WDM systems and will bring about a new way for designing topological photonic devices.

## 2. Materials and Methods

**Figure 1:**
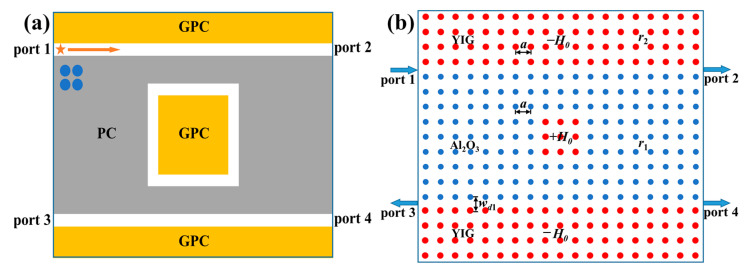
The topological four-port ADF: (**a**) schematic diagram; (**b**) illustration in a 2D PC structure. The orange and gray rectangles represent the GPC and the dielectric PC respectively, and the yellow arrow indicates the transport direction of TES. The blue and red circles denote Al_2_O_3_ rods and YIG rods, respectively.

Figure 1a shows a schematic diagram of the proposed four-port ADF. It is composed of two GPC waveguides and a nested square PCRR sandwiched between them. The device consisted of four ports: port 1 (the input terminal), port 2 (the through terminal), port 3 (the backward dropping terminal), and port 4 (the forward dropping terminal). The upper and lower waveguides named as bus waveguide and dropping waveguide were placed symmetrically with respect to the PCRR, and they were constructed by two GPCs (orange rectangle) and a dielectric PC (gray rectangle). 

For the topological four-port ADF studied here, we utilized coupled mode theory to investigate the interaction between GPC waveguide and the PCRR. The main idea of coupled mode theory is to analyze the coupling characteristics between the ring resonator and waveguide by solving the time domain differential equation of the input and output amplitude field components [48]. A 2D point source (yellow star) was placed near the input port 1; when ignoring the dispersion loss of waveguides and internal loss of the PCRR, the coupled mode equation in the time domain can be expressed as [49]

(1) $$\frac{da}{dt}=\left(j2\pi f_{0}-\frac{2}{\tau }\right)a+\sqrt{\frac{2}{\tau }}e^{j\theta }S_{1}$$

where *a* is the normalized amplitude of the resonant cavity, *f*_0_ is the resonant frequency, 1/*τ* is the decay rate coupled into each waveguide, *S_i_* (*i* = 1, 2, 3, 4) is the normalized amplitude of input (output) wave, and *θ* represents the phase of the coupling coefficient from input wave to the cavity mode. 

When the EMFs applied to the upper and lower GPC arrays are in the −*z* direction, both the bus and the dropping waveguide are nonreciprocal. According to the conservation of energy and one-way transmission principle, the amplitude of output waves should satisfy the following relationships:

(2) $$S_{2}=S_{1}-\sqrt{\frac{2}{\tau }}e^{-j\theta }a\;S_{3}=-\sqrt{\frac{2}{\tau }}e^{-j\theta }a\;S_{4}=0$$

The transmittance of the three output ports is

(3) $$T_{2}={\left|\frac{S_{2}}{S_{1}}\right|}^{2}={\left|1-\frac{\frac{2}{\tau }}{j2\pi (f-f_{0})+\frac{2}{\tau }}\right|}^{2}$$

(4) $$T_{3}={\left|\frac{-S_{3}}{S_{1}}\right|}^{2}={\left|\frac{\frac{2}{\tau }}{j2\pi (f-f_{0})+\frac{2}{\tau }}\right|}^{2}$$

(5) $$T_{4}={\left|\frac{S_{4}}{S_{1}}\right|}^{2}=0$$

As can be seen from the above formulas, *T*_4_ = 0 due to one-way TES, *T*_2_ = 0 and *T*_3_ = 1 at the resonant frequency *f*_0_, meaning that the maximum transmission efficiency is achieved, and the phase difference meets *θ* = 2*nπ* (*n* is an integer) at resonance. Thus, when we select the appropriate position and size of the PCRR to make the phase difference equal to *θ* = 2*nπ*, constructive interference of light waves occurs to realize 100% coupling output. It should be noted that if the propagation direction in the dropping waveguide is reversed, the above equations are still valid, but the subscripts ‘3’ and ‘4’ need to be exchanged. In this case, port 4 achieves a 100% throughout, while there is no output at port 3.

The concrete PC structure implementing the aforementioned topological four-port ADF is drawn in Figure 1b. Here, the dielectric PC (in blue) and the GPC (in red) used in the simulation were constructed by alumina (Al_2_O_3_) rods and yttrium–iron–garnet (YIG) rods, respectively. In order to satisfy constructive interference of EM waves for efficient coupling, the coupling distances between the PCRR and two waveguides were set to be three rows of Al_2_O_3_ rods, and the square PCRR was formed by a 3 × 3 YIG rods. Both the Al_2_O_3_ PC and the GPC were immersed in air and arranged in a square lattice with the same lattice constant, i.e., *a* = 3.87 cm. Their radii were *r*_1_ = 0.13*a* and *r*_2_ = 0.15*a*, respectively, and their relative permittivities were *ε*_1_ = 10 and *ε*_2_ = 15, respectively. The widths of two topological waveguides were the same, being *w_d_*_1_ = *a*. The permeability of YIG rods was 1 in the absence of EMF. When a *dc* magnetic field *H*_0_ was applied along the out-of-plane (+*z*) direction, a strong gyromagnetic anisotropy was induced in YIG rods with the permeability becoming a tensor as follows:

(6) $$\hat{\mu }=\left(\begin{array}{ccc} \mu_{r} & j\mu_{k} & 0\\ -j\mu_{k} & \mu_{r} & 0\\ 0 & 0 & 1 \end{array}\right)$$

where $\mu_{r}=1+\omega_{m}\left(\omega_{0}+j\alpha \omega \right)/[{\left(\omega_{0}+j\alpha \omega \right)}^{2}-\omega^{2}]$ and $\mu_{k}=\omega \omega_{m}[{\left(\omega_{0}+j\alpha \omega \right)}^{2}-\omega^{2}]$, with *ω*_0_ = 2π*γH*_0_ being the resonance frequency, *γ* = 2.8 MHz/Oe being the gyromagnetic ratio, and *ω_m_* = 2π*γM*_0_ being the characteristic circular frequency, where *M*_0_ = 1780 G is the saturation magnetization. The applied magnetic field was set to *H*_0_ = 1600 G, and the damping coefficient was ignored (*α* = 0). Additionally, all numerical simulations were conducted by using the Finite Element Method, and only the E polarization (where the electric field E is parallel to the *z*-axis direction) was considered.

It is known that the Chern number is defined as the integral of the Berry flux over the entire Brillouin zone, and calculation of the Chern number requires discretization of the first Brillouin zone in the wave-vector *K* space [50,51]. The Chern number of the *n*th band is defined as

(7) $$C_{n}=\frac{1}{2\pi i}\int_{BZ}\left(\frac{\partial A_{y}^{nn}}{\partial k_{x}}-\frac{\partial A_{x}^{nn}}{\partial k_{y}}\right)d^{2}k$$

Here, the wave-vector space is varying over the first Brillouin zone, and the Berry connection is given by $A^{nn}(k)=i\langle E_{nk}\left|\nabla_{k}\right|E_{nk}\rangle$, where ***E****_nk_* denotes the normalized eigenstate for the *n*th band at *k* point and is the periodic part of the electric-field Bloch function. The Chern number can be utilized to predict the existence of TESs and determine their number in a 2D GPC structure. A general way to calculate the gap Chern number (*C_gap_* = ∑*C_n_*) is to sum the Chern numbers of all bands below the bandgap; trivial and topologically nontrivial have zero (*C_gap_* = 0) and nonzero (*C_gap_* ≠ 0) Chern numbers’ bandgaps, respectively [52,53]. 

To numerically solve the gap Chern number of GPC and Al_2_O_3_ PC, we calculated the band structures by employing the Finite Element Method. The results showed that the gap Chern number of Al_2_O_3_ PC was 0, and thus the bandgap was trivial. Conversely, the gap Chern number in the second bandgap (between the second and third bands) of YIG PC was +1(or −1) when the EMF was −*H*_0_(or +*H*_0_), which revealed that this bandgap was topologically nontrivial [54,55]. On the other hand, because the bandgap of Al_2_O_3_ PC overlapped the second bandgap of YIG PC, the number of topological one-way edge states in the waveguide composed of these two PCs was equal to the difference of gap Chern numbers, and the propagation direction of topological one-way edge states was determined by the sign of gap Chern numbers. It can be easily calculated that the Chern difference Δ*C_gap_* of the domain wall between Al_2_O_3_ PC (*C_gap_* = 0) and YIG PC (*C_gap_* = ±1) was +1 or −1, indicating that this waveguide supported only a one-way TES that was topologically protected.

**Figure 2:**
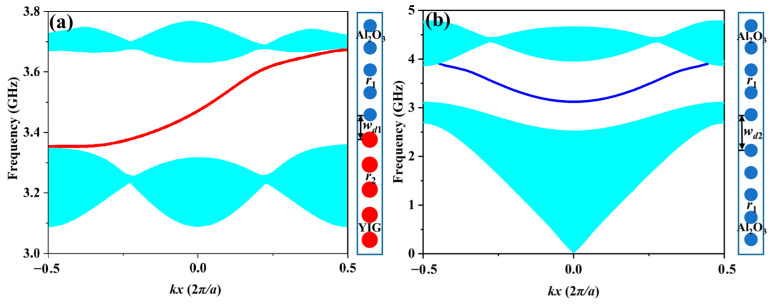
Projected band diagrams of different types of waveguides: (**a**) one-way waveguide; (**b**) ordinary Al_2_O_3_ PC waveguide. The red and blue curves represent the corresponding waveguide modes, and the cyan areas are the bulk band regions.

Next, we calculated the projected band structure of the transverse magnetic (TM) mode with +*H*_0_ applied to YIG PC by adopting a supercell consisting of Al_2_O_3_ and YIG rods in one column, as depicted in Figure 2a. It can be clearly seen that a red dispersion curve appeared in the bandgap ranging from 3.35 to 3.65 GHz and connected the upper and lower bands. Moreover, the slope of the red curve was always positive, meaning the waveguide supported a topological one-way edge state propagating rightwards, which was in good agreement with the theoretical prediction by the Chern number. It was noted that propagation direction of EM waves in the one-way waveguide was dependent on the EMF applied to the GPC. Once the sign of the EMF was reversed, the propagation was switched to the opposite direction accordingly. In addition, we also analyzed the projected band diagram of the ordinary square Al_2_O_3_ PC waveguide. In order to match the bandgap of one-way waveguide shown in Figure 2a, the width of the Al_2_O_3_ PC waveguide was set to *w_d_*_2_ = 1.5*a*. One can see from Figure 2b that a blue dispersion curve appeared in the bandgap ranging from 3.2 to 3.9 GHz that was symmetrical at about *k_x_* = 0, indicating that the ordinary PC waveguide supported bidirectional transmission and was not topologically protected because of the trivial bandgap. 

## 3. Results

### 3.1. Topological Four-Port ADF

**Figure 3:**
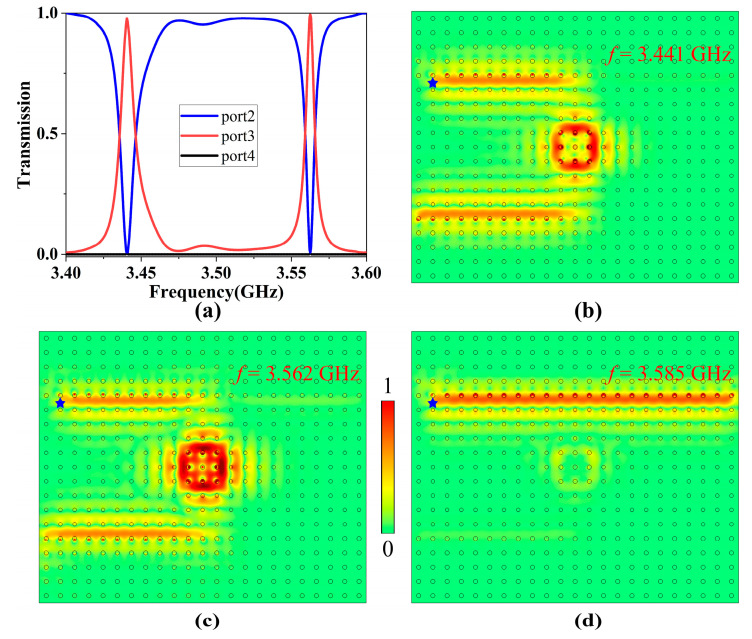
(**a**) The normalized transmission spectra of three output ports when *H*_0_ = 1600 G. (**b**–**d**) The electric field distributions at 3.441, 3.562, and 3.585 GHz, respectively. The blue stars represent the point sources.

Firstly, we studied the transmission characteristics of the topological four-port ADF. We numerically simulated the transmission behaviors of the topological one-way filter. The scattering boundary conditions were placed around the structure to avoid the reflection, a 2D point source (blue star) was placed near the input port 1, and boundary probes were placed at three other output ports to measure their power outputs. The normalized transmission spectra of three output ports were calculated and are shown in Figure 3a. One can see that there were two transmission peaks for port 3 at frequencies 3.441 GHz and 3.562 GHz, with transmittances as high as 98.6% and 99.5%, respectively, and insertion losses about 0.223 dB and 0.206 dB, respectively. Meanwhile, the transmittance of port 2 decreased to zero at these two frequencies. Moreover, the transmittance of port 4 in the whole frequency range remained at zero, meaning that there was no energy output. The appearance of these two resonant modes also verified the multimode characteristics of the square PCRR.

In order to better observe the filtering effect of the ADF, we further simulated the electric field distributions for incident EM waves at three different frequencies, as shown in Figure 3b–d. It was clearly seen that EM waves oscillating at the resonant frequencies of 3.441 GHz and 3.562 GHz satisfied the resonant coupling condition, and they were able to enter the ring resonator and be coupled to the dropping waveguide efficiently; conversely, EM waves at other frequencies (such as 3.585 GHz) did not meet the resonant coupling condition, and thus they were only able to travel to port 2 instead of entering the PCRR. Therefore, the resonant coupling phenomenon enabled EM waves at resonant frequencies to transmit from the bus waveguide to the dropping waveguide with almost no energy loss, achieving efficient filtering function. It is worth noting that when we reversed the direction of EMF applied to the GPC next to the dropping waveguide, EM waves at resonant frequencies would exit from port 4 instead of port 3, and thus the output port could be switched dynamically.

Our simulation results showed that the lattice constant *a* and the radius of YIG rods *r*_2_ had effects on the resonant frequencies and the bandgap range, respectively. Specifically, when the lattice constant decreased (or increased), the resonant frequencies were blue-shifted (or red-shifted), and when the radius of YIG rods decreased (or increases), the bandgap range of the one-way waveguide was blue-shifted (or red-shifted). To ensure good performance of the topological ADF, the permissible error in the lattice constant and the size of the YIG rods should be less than ±0.01 cm and ±0.01*a*, respectively. Otherwise, the resonant frequencies will deviate, and the common bandgaps of two PCs will become smaller or even disappear, which not only reduces the operating frequency range of the topological ADF but also significantly affects the transmission efficiency.

**Figure 4:**
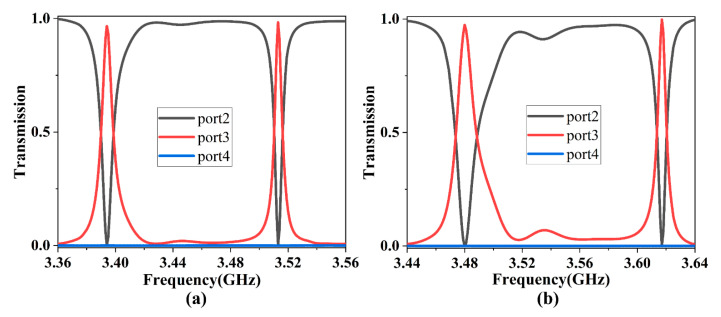
The normalized transmission spectra when different intensities of EMFs were applied to the PCRR: (**a**) 1500 G; (**b**) 1700 G.

We further discuss the influence of the EMF intensity *H*_0_ applied to the square PCRR on the transmission performance of the ADF. When the EMF intensity decreased to *H*_0_ = 1500 G (keeping other structural parameters the same as those in Figure 1), the corresponding transmission spectra of three output ports were as shown in Figure 4a. There were two transmission peaks at 3.394 GHz and 3.513 GHz, with transmittances of 97.2% and 98.6%, respectively. When the EMF intensity increased to *H*_0_ = 1700 G, the corresponding transmission spectra were as shown in Figure 4b. There were also two transmission peaks at 3.480 GHz and 3.617 GHz, with transmittances of 97.4% and 99.8%, respectively.

Combined with the analysis in Figure 3a, when the EMF intensity *H*_0_ increased from 1500 to 1700 G, the ADF always had two resonant modes and maintained high transmittance. Additionally, the overall transmission spectra moved towards higher frequency, and the corresponding resonant frequencies were blue-shifted. Therefore, without changing structure parameters, we were able to adjust the resonant frequencies by only changing the EMF intensity to achieve a tunable ADF. 

### 3.2. Topological Multichannel ADF

On the basis of the nonreciprocity, topological protection, and flexible configurations of the four-port ADF, we further designed a topological multichannel ADF by paralleling two square PCRRs to realize more unique functions. These two PCRRs share a middle ordinary PC waveguide and have the same dimensions and parameters. Such multichannel ADF can work as a power splitter or a demultiplexer under different conditions.

**Figure 5:**
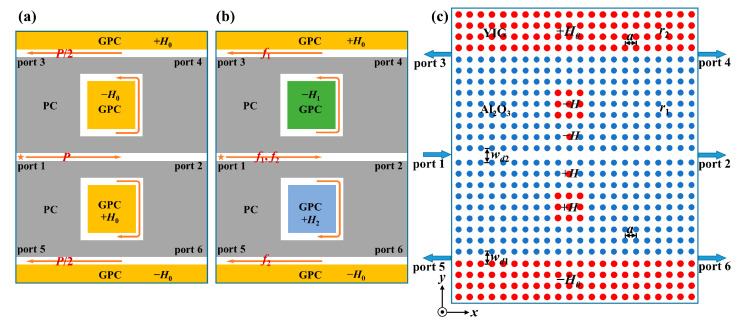
Schematic diagrams of the multichannel ADF in different modes: (**a**) power splitting mode; (**b**) frequency separating mode. (**c**) Illustration of the multichannel ADF in a PC structure. The orange, green and blue rectangles represent the GPC with different EMFs, and the yellow arrows indicate the transport directions of energy fluxes in different modes. The blue and red circles denote Al_2_O_3_ rods and YIG rods, respectively.

Figure 5a,b depicts the schematic diagrams of power splitting mode and frequency separating mode for the topological multichannel ADF, respectively. As shown in Figure 5a, when the intensities of EMFs for the two square PCRRs were the same (i.e., −*H*_0_ and +*H*_0_), the corresponding resonant modes of both PCRRs were also the same. In this case, when EM wave oscillating at the resonant frequency with power of *P* is incident from the middle ordinary PC waveguide, it will reach the upper and lower output waveguides with the same transmission efficiency via the coupling effect of these two PCRRs. The energy will be split into two equal parts (i.e., *P*/2) to the corresponding output ports, thus realizing the function of power division. Differently, as shown in Figure 5b, when the intensities of EMFs for the two square PCRRs were different (i.e., −*H*_1_ and +*H*_2_), the corresponding resonant modes of these two PCRRs were different from each. In this case, when two EM waves oscillating at two different resonant frequencies (i.e., *f*_1_ and *f*_2_) are incident from the middle ordinary waveguide simultaneously, they will reach different output waveguides through the coupling effect of these two PCRRs, thus realizing the function of frequency separation. Therefore, without changing structural parameters of the multichannel ADF, the functions of power division and frequency separation can be achieved by flexibly adjusting the intensities of EMFs applied to the two square PCRRs.

The concrete PC structure implementing the topological multichannel ADF is illustrated in Figure 5c. The multichannel filter had six ports. The middle main channel was an Al_2_O_3_ PC waveguide with a width of *w_d_*_2_ = 1.5*a*, and the upper and lower output channels were one-way waveguides with the same width of *w_d_*_1_ = 1*a*. Two square PCRRs with the same structural parameters were symmetrically distributed on both sides of the main channel. Moreover, we also introduced a point-defect microcavity between the main channel and each PCRR to further improve transmission efficiency. These two microcavities were introduced by replacing two symmetrical Al_2_O_3_ rods with two YIG rods that had the same structural parameters and EMFs as those of the adjacent PCRRs. The resonator system composed of the point-defect microcavity and the PCRR not only had a strong electric field localization ability but also can further filter out redundant frequencies.

As for the practical experiment, it required YIG and Al_2_O_3_ cylinders, magnets, a microwave network analyzer, metal plates, and a computer. The key issue was the way in which to apply the EMFs to different parts of the YIG cylinders of the structure. Two metal plates were set in the upper and lower parts of the designed structure, and they were also carved out with hole arrays (corresponding to the YIG cylinders) to place magnet arrays, but they were not completely hollowed out and left a certain thickness in order to forbid the EM waves to transport in the *z* direction. A pair of magnet arrays were pressed into the holes in metal plates above and below the YIG structure, respectively, and the positive and negative magnetic fields were produced by the different combinations of the magnetic poles, respectively. This new magnetization scheme not only can accurately magnetize each YIG cylinder but also can freely adjust the direction and intensity of the EMFs.

#### 3.2.1. Power Splitting Mode

**Figure 6:**
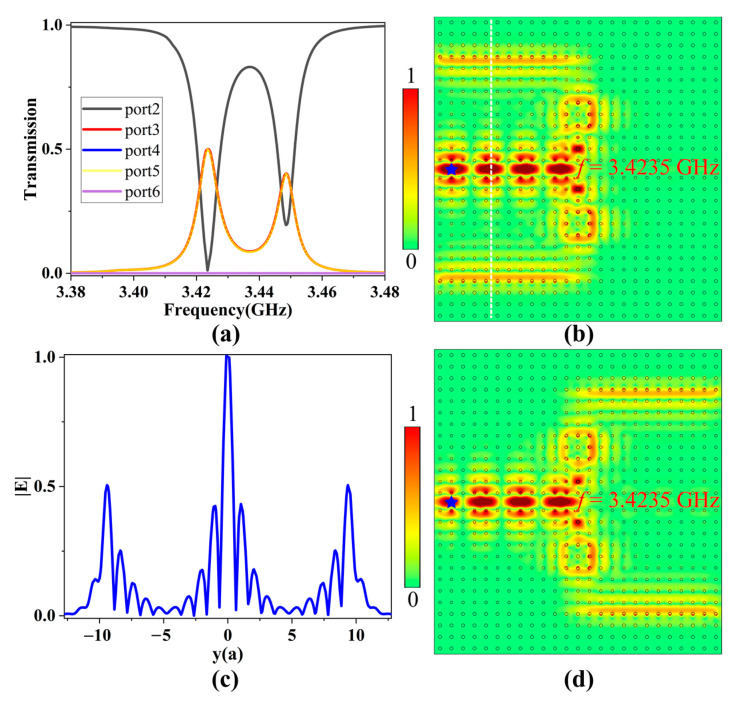
(**a**) The transmission spectrum when the EMF intensity of two resonator systems was 1600 G. (**b**) The electric field distribution at the resonant frequency. (**c**) The normalized electric field amplitude distributions along the vertical white dotted line. (**d**) The electric field distribution at the resonant frequency when the upper and lower EMFs were reversed. The blue stars represent the point sources.

When the two resonator systems were applied with the same intensity of EMF (i.e., *H*_0_ = 1600 G), due to the strong coupling and filtering effect between point-defect microcavity and the PCRR, they had the same resonant modes. A 2D point source was placed near the input port 1, and boundary probes were added to the other five output ports to calculate their power outputs. The transmission spectra of ports 2–6 from 3.38 to 3.48 GHz are shown in Figure 6a. Obviously, ports 3 and 5 had two same transmission peaks, and they had a transmittance of 50% only at 3.4235 GHz; conversely, the transmittance of port 2 decreased to zero, and the transmittances of ports 4 and 6 were always zero. Thus, due to the resonant coupling effect of two identical resonator systems, EM wave at the resonant frequency in the main channel was equally divided into two parts, then coupled into two one-way waveguides and finally output from the corresponding ports.

The electric field distribution in a multichannel filter at 3.4235 GHz is shown in Figure 6b. The EM wave incident from port 1 was split into two equal parts because of the coupling effect of the two resonant systems, and finally they exited from ports 3 and 5, respectively. In order to obtain the power division ratio, we calculated the normalized electric field amplitude distributions along the vertical white dotted line. As seen in Figure 6c, the power values for ports 3 and 5 were about 50% of that in the main channel, which was in good agreement with the transmission spectra. As a result, the function of power division was realized. Furthermore, when the directions of the EMFs applied to the upper and lower GPCs were reversed simultaneously, the direction of the TESs supported in two one-way waveguides was reversed accordingly, as plotted in Figure 6d. In this case, ports 4 and 6 became output ports with the same energy distribution and high transmittance, which allowed us to dynamically switch the output ports of the multichannel filter. Therefore, when two resonator systems were applied with the same EMF intensity, the multichannel ADF was equivalent to a power splitter.

#### 3.2.2. Frequency Separating Mode

**Figure 7:**
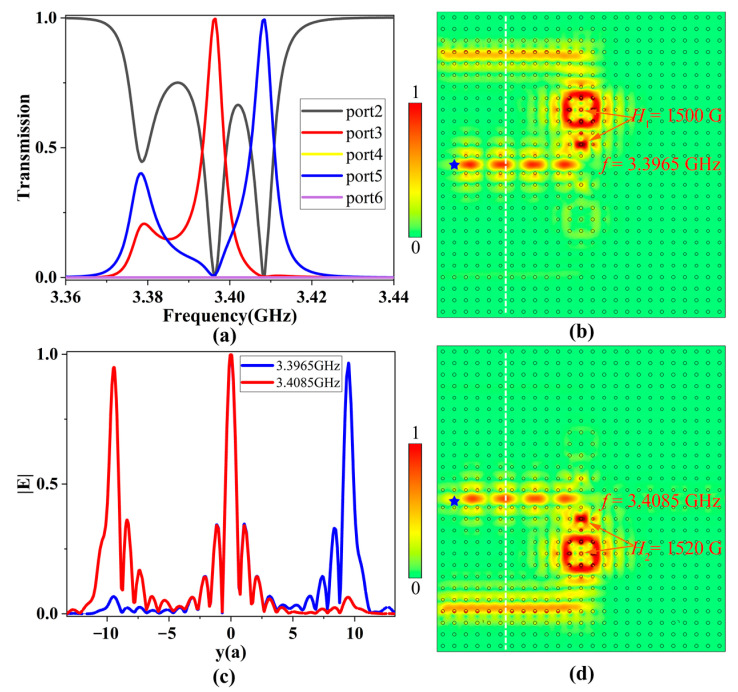
(**a**) The transmission spectra when the EMF intensities of two resonator systems were 1500 G and 1520 G, respectively. (**b**) The electric field distribution at 3.3965 GHz. (**c**) The normalized electric field amplitude distributions along the vertical white dotted line. (**d**) The electric field distribution at 3.4085 GHz. The blue stars represent the point sources.

When the two resonator systems are applied with different magnetic field intensities (i.e., *H*_1_ = 1500 G and *H*_2_ = 1520 G respectively), they will have different resonant modes. EM waves with frequencies ranging from 3.36 to 3.44 GHz are incident from port 1, and the transmission spectra of the five output ports 2–5 are shown in Figure 7a. One can find that the transmission peaks of ports 3 and 5 were obtained at 3.3965 GHz and 3.4085 GHz with transmittances of 99.6% and 99.5%, respectively. Conversely, the transmittance of port 2 decreased to zero at both resonant frequencies, and the transmittances of ports 4 and 6 were always zero. Therefore, by changing the intensities of EMFs for the two resonator systems, these two resonator systems will have their own resonant modes to effectively separate two different resonant frequencies.

The electric field distributions at 3.3965 and 3.4085 GHz are illustrated in Figure 7b,d, respectively. It is clearly seen that EM waves at different resonant frequencies were efficiently coupled to the corresponding PCRR, and then transferred to different output channels with little energy loss. Specifically, EM wave at 3.3965 GHz can only couple with the upper resonator and then travel to port 3, while that at 3.4085 GHz can only couple with the lower resonator and then travel to port 5, which is in good agreement with the results of the transmission spectra. To observe the energy distributions in the three channels for these two resonant modes, we calculated the normalized electric field amplitude distributions along the vertical white dotted lines, as shown in Figure 7c. For these two resonant modes, almost all incident EM wave energy in the main channel was delivered to the corresponding output channel, indicating that each channel worked independently and did not interfere with each other during the transmission process, ensuring high channel isolation for the multichannel ADF. Therefore, when two resonator systems are applied with different intensities of EMFs, the topological multichannel filter is equivalent to a demultiplexer.

#### 3.2.3. Robustness Analysis

Finally, we investigated the influence of two different kinds of defects on the multichannel ADF to verify its robustness. One is to insert two perfect electrical conductors (PECs) with a size of 0.1*a* × 2*a* (represented by white rectangles) into the output waveguides, while the other is to shift up a YIG rod in the PCRR by 0.15*a* (circled in cyan squares). All other structural parameters are the same as those in Figure 5.

**Figure 8:**
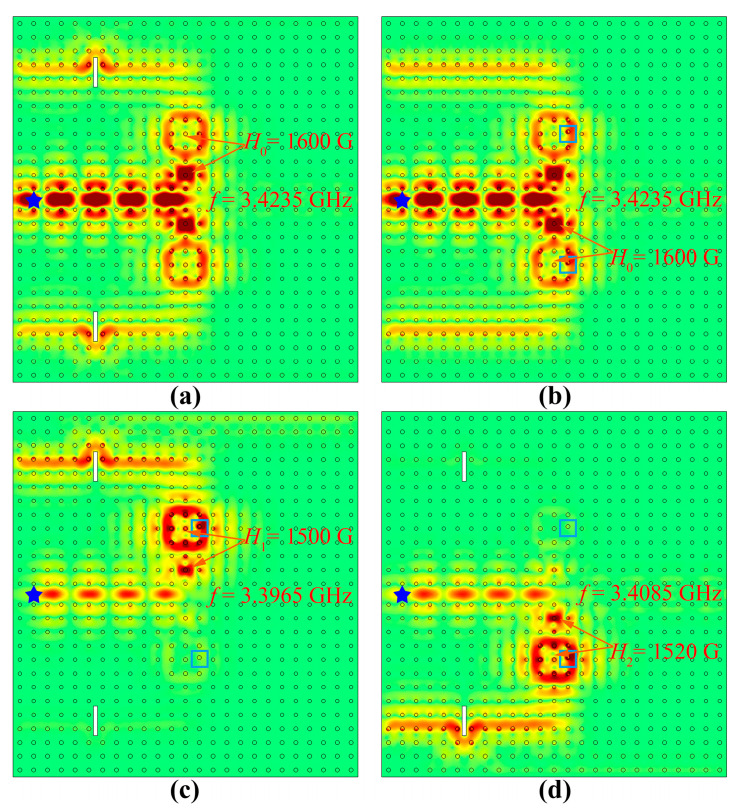
The electric field distributions in power splitting mode (**a**,**b**) and frequency separating mode (**c**,**d**): (**a**) with PECs in the waveguides; (**b**) with disorders in the PCRRs; (**c**) with both PECs and disorders at 3.3965 GHz; (**d**) with both PECs and disorders at 3.4085 GHz. The white rectangles and cyan squares represent the inserted PECs and the position movement of YIG rods respectively, The PECs and disorders are denoted by white rectangles and cyan squares, respectively, and the blue starts denote the point sources.

For the power splitting mode, we studied the transmission behaviors of the multichannel ADF when different defects were introduced. We found that the transmission spectra were the same as those without any defects shown in Figure 6a. The corresponding electric field distributions at the resonant frequency 3.4235 GHz are illustrated in Figure 8a,b, which were almost unchanged before and after these defects. For the frequency separating mode, we studied the transmission behaviors of the multichannel ADF when both PECs and disorder defects were introduced simultaneously for the two resonant frequencies of 3.3965 GHz and 3.4085 GHz. It was found that the transmission spectra were also the same as those shown in Figure 7a, and there still were two transmittance peaks for ports 3 and 5 at 3.3965 GHz and 3.4085 GHz, respectively. The corresponding electric field distributions are illustrated in Figure 8c,d, which were almost the same as those shown in Figure 7b,d. In both working modes, due to the existence of TESs, EM waves can circumvent these defects instead of being blocked and continue to propagate unidirectionally. Although these defects cause a phase retardation, the transmission efficiency remains unaffected. These results imply that the multichannel ADF has strong robustness, which provides good fabrication tolerance and high performance for the ADF.

## 4. Conclusions

In conclusion, we theoretically proposed and numerically investigated a new configuration of multichannel ADF by coupling the topological PCRR with one-way GPC waveguides. We first proposed a topological four-port ADF composed of two one-way waveguides and a square PCRR, finding that the resonant frequencies can be controlled by adjusting the EMF intensity without changing structure parameters. Next, we further designed a topological multichannel ADF by paralleling two square PCRRs, able to realize the functions of power division and frequency separation by adjusting the intensities of EMFs in the resonator systems. Not only can this device possess excellent filtering performance, it also can tolerate various defects such as the PECs and disorders due to the topological protection property. Moreover, each output port can be switched dynamically, and each transmission channel can operate independently with little crosstalk. These results have the potential for developing topological photonic devices with higher performance in WDM systems.

## Data Availability

The data presented in this study are available on request from the corresponding author.
